# Green synthesis of MnO_2_ NPs using Arabic gum: assessing its potential antiviral activity against influenza A/H1N1

**DOI:** 10.1186/s12985-024-02315-z

**Published:** 2024-02-23

**Authors:** Neda Baghban, Safieh Momeni, Emad Behboudi, Hassan Dianat-Moghadam, Amirhossein Darabi, Hadiseh Shokouhi Targhi, Mohsen Keshavarz

**Affiliations:** 1 The Persian Gulf Marine Biotechnology Research Center, The Persian Gulf Biomedical Sciences Research Institute, Bushehr University of Medical Sciences, Bushehr, Iran; 2 Department of Basic Medical Sciences, Khoy University of Medical Sciences, Khoy, Iran; 3 Department of Genetics and Molecular Biology, School of Medicine, Isfahan University of Medical Sciences, Isfahan, Iran; 4 Pediatric Inherited Diseases Research Center, Isfahan University of Medical Sciences, Isfahan, Iran; 5 The Persian Gulf Tropical Medicine Research Center, The Persian Gulf Biomedical Sciences Research Institute, Bushehr University of Medical Sciences, Bushehr, Iran; 6 Influenza and Respiratory Viruses Department, Pasteur Institute of Iran, Tehran, Iran

**Keywords:** MnO_2_, Arabic gum, Influenza, Nanoparticle, Antiviral

## Abstract

**Background:**

The antiviral properties of metal nanoparticles against various viruses, including those resistant to drugs, are currently a subject of intensive research. Recently, the green synthesis of nanoparticles and their anti-viral function have attracted a lot of attention. Previous studies have shown promising results in the use of Arabic gum for the green synthesis of nanoparticles with strong antiviral properties. In this study we aimed to investigate the antiviral effects of MnO_2_ nanoparticles (MnO_2_-NPs) synthesized using Arabic gum, particularly against the influenza virus.

**Methods:**

Arabic gum was used as a natural polymer to extract and synthesize MnO_2_-NPs using a green chemistry approach. The synthesized MnO_2_-NPs were characterized using SEM and TEM. To evaluate virus titration, cytotoxicity, and antiviral activity, TCID50, MTT, and Hemagglutination assay (HA) were performed, respectively. Molecular docking studies were also performed to investigate the potential antiviral activity of the synthesized MnO_2_-NPs against the influenza virus. The molecular docking was carried out using AutoDock Vina software followed by an analysis with VMD software to investigate the interaction between Arabic gum and the hemagglutinin protein.

**Results:**

Simultaneous combination treatment with the green-synthesized MnO_2_-NPs resulted in a 3.5 log HA decrement and 69.7% cellular protection, which demonstrated the most significant difference in cellular protection compared to the virus control group (*p*-value < 0.01). The docking results showed that binding affinities were between − 3.3 and − 5.8 kcal/mole relating with the interaction between target with MnO_2_ and beta-D-galactopyranuronic acid, respectively.

**Conclusion:**

The results of the study indicated that the MnO_2_-NPs synthesized with Arabic gum had significant antiviral effects against the influenza virus, highlighting their potential as a natural and effective treatment for inhibition of respiratory infections.

## Introduction

The influenza virus, a prevalent respiratory pathogen with genetic variation and immune evasion, continues to pose a formidable challenge to scientists [1]. Antigenic drift, which is small changes in hemagglutinin (HA) or neuraminidase (NA) amino acids, enables the virus to evade the immune system. The primary mode of transmission for the influenza virus is airborne droplets released during speaking, coughing or sneezing by infected individuals [2]. Since 1900, three major influenza pandemics have been documented [3]. Seasonal influenza epidemics typically occur annually, particularly during winter or beginning of spring. This seasonal pattern seems to have several reasons, including constant drifts in the virus each season and behavioral factors [4, 5]. In 1960s amantadine and rimantadine as first antiviral agents were shown to have prophylactic and therapeutic activities for influenza A [1]. Although there are several known vaccines and antivirals including M2 and neuraminidase inhibitors that have been developed and approved for influenza treatment, drug resistance and delivery routes cause significant limitations to the antivirals and seasonal influenza is still a big public health problem [6].

These limitations have led scientists to other antiviral agents and among various antiviral strategies, nanotechnology seemed to have the ability to address the problems and many nanoparticles (NPs) have been reported for their positively effect on virus infections and replication [7–10]. Nanotechnology has been shown to be useful in different aspects of virus research from various biosensors produced by nanotechnology-based probes to the research of molecular mechanisms of virus-infected cells. Furthermore, the antimicrobial potential of metal NPs against a variety of viral pathogens including drug resistant viruses have been investigated before [11]. NPs can exert their antiviral activity in a variety of ways, such as binding to the virus surface to inactivate DNA/RNA function, penetrating host cells to destroy its structure, and producing reactive oxygen species (ROS) [12, 13]. In the past two decades, nanomaterials have been extensively researched for drug delivery application and have been improved for their physiochemical and therapeutic effects.

Chemical, physical and biological characteristics of NPs such as surface area, size, distribution, and morphology indicate their antimicrobial effects [14–16]. Previous investigations have shown that the antimicrobial effects of NPs depend on their synthesis method, which can be classified into physical, chemical and biological methods. Among them physical methods have the highest costs in the aspects of economics and energetics because it requires expensive equipment, high temperature, and high pressure which makes it an unprofitable method [17, 18]. Chemical methods also are not a perfect solution since they might be cytotoxic, carcinogenic, or harmful for the environment [19]. However, the biological method, known as “green synthesis”, which uses microorganisms and plants to produce NPs, undoubtedly has fewer environmental impacts and offers other advantages, including safety, efficiency, and profitability. Recently, natural flavonoids have emerged as a promising category of antiviral compounds [20]. However, these flavonoids suffer from poor solubility and rapid degradation by metabolism [21]. To overcome these limitations, drug delivery systems and green synthesized nanoparticle-based approaches can modify flavonoids, allowing for long half-life, high affinity binding, and strong solubility. The incorporation of herbal drugs into the delivery system can also enhance stability, tissue macrophage distribution, pharmacological activity, sustained delivery, bioavailability, and targeting ability of flavonoids [22]. Arabic gum, a natural polymer consisting hydrophile polysaccharides and hydrophobe proteins, has been previously investigated for the green synthesis of NPs and their antiviral effects, showing significant potential in antiviral aspects [23].

In this study, our aim was to investigate the antiviral effects of green-synthesized MnO_2_-NPs, extracted using Arabic gum, with a focus on the influenza virus.

## Methods

### Preparation and characterization of MnO_2_

We obtained various chemical reagents with analytical purity, including Potassium permanganate (KMnO_4_), Sodium hydroxide (NaOH) and ethanol, from their respective sources. Arabic Gum was supplied by Sigma-Aldrich. Deionized water was used to prepare the aqueous solution for all experiments. The measurements were taken under a pressure of 1 atmosphere and at a temperature of 25^o^C.

In terms of apparatus, the surface morphology of the nanomaterials was studied using field emission electron microscopy (Hitachi S-4160 FESEM), while transmission electron microscopy images of the MnO_2_ nanosheets were obtained using JEOL JEM-2100T, operating at 200 kV.

To green synthesize MnO_2_-NPs, Arabic Gum (100 mg) was dissolved in 20 mL of deionized water, followed by the addition of KMnO_4_ solution (1.5 mg/mL, 20 mL) with vigorous stirring. The solution was then heated at 50 °C for 5 h, during which the color of the solution changed from purple to brown, indicating the formation of MnO_2_-NPs. The formation of MnO_2_ nanosheets was confirmed by various characterization methods. After the synthesis, the MnO_2_-NPs were collected, and the pH of the suspension was carefully adjusted to a physiological pH (7.4) using a Metrohm pH meter (model 780).

### Cell culture and virus propagation

Madin-Darby canine kidney cells were purchased from the Pasteur institute of Iran, Tehran. The cells were grown at 37 °C in 5% CO_2_ with 85% of humidity in DMEM(BIO-IDEA), supplemented with 10% of Fetal Bovine serum (DNABIOTECH) and 1% of penicillin (BIO-IDEA). The influenza virus vaccine strain, A/Puerto Rico/8/1934 (H1N1) (ATCC VR-897™), sourced from the Influenza Department at the Pasteur Institute of Iran, was cultured in MDCK cells. The virus was cultured in MDCK cells with the addition of 1 μg/ml of trypsin-Tosylamide Phenylethyl Chloromethyl Keton-treated Trypsin (TPCK) from Sigma, USA. After a 48-hour incubation period, the supernatant containing the virus was collected and the Reed and Muench formula was used for virus titration [24].

### Determination of cell viability

Methyl thiazolyl tetrazolium (MTT) assay was used to assess the cytotoxicity of the green-synthesized MnO_2_-NPs on MDCK cells. A 96-well plate was used to seed MDCK cells for 24 h and incubate at 37 °C in 5% CO_2_. Then, a variety of NP concentrations, prepared by 2-fold serial dilutions, were added to the wells in triplicate. After two days of incubation, the medium containing the green-synthesized MnO_2_ was removed, and MTT reagent (5 mg/mL) was added to the wells, followed by incubation in the dark for another 4 h. Then, the medium was discarded and 100 μL of DMSO was added to each well and the plate was shaken for 5 min in RT to dissolve the formazan crystals. Afterwards, the plate was read at 470 nm using an ELISA reader.

### Assessment of antiviral activity

Three separate 96-well plates were seeded with MDCK cells and incubated for 24 h to assess the antiviral activity of green-synthesized MnO_2_-NPs in pre-penetration, co-penetration, and post-penetration treatments. For both pre- and post-penetration procedures, 100 μl of non-cytotoxic concentration (NCTC) of the green-synthesized MnO_2_ was added to the wells before and after the virus concentrations, followed by a one-hour incubation. Subsequently, the wells were washed with PBS, and the virus (100TCID50) was introduced, leading to another hour of incubation. After this incubation period, the wells were washed again, and 100 μL of TPCK-containing DMEM with 3% FBS was added, followed by incubation for 3 days at 37 °C in 5% CO_2_ with 85% humidity.

For co-penetration, 100 μl of NCTC of the green-synthesized MnO_2_ was mixed with the influenza virus (100TCID50), incubated for 30 min, and then added to the wells. This was followed by an additional one-hour incubation. All three procedures were conducted in triplicate wells and were subsequently assessed using the MTT assay to evaluate cellular protection, as described in the MTT process. Concurrently, the hemagglutination assay (HA) was performed to determine the virus titer in the cell supernatants. Control antiviral drugs used included amantadine hydrochloride and oseltamivir carboxylate.

### Percentage of protection

Cell viability for both infected and non-infected cells was determined based on formazan absorbance values. The percentage of protection was computed using the following formula:

Percentage of protection = [A-B] / [C-B] ×100.

where A, B, and C represent the absorbance of the sample, the virus-infected control (without any compound), and the mock-infected control (without virus or compound), respectively [25].

### Molecular docking

The molecular docking software Autodock Vina 1.1.2 was employed to locate the exact binding site of the ligand on the protein. The three-dimensional (3D) structures of seven components and the Influenza A virus (A/Puerto Rico/8/1934 (H1N1)), with PDB code of 6wcr (key target), were obtained from the PUBCHEM and PDB databases, respectively. The selection of the hemagglutinin protein (HA) from the Influenza A virus (PDB code 6wcr) is based on the critical role of HA in viral entry, making it a relevant target for understanding the potential antiviral activity of the synthesized MnO_2_-NPs. The choice is informed by the need to disrupt the interaction between the virus and host cells, which could lead to effective antiviral strategies. To modify structures for docking analysis, Chimera was applied. In order to fit targets for docking analysis, outlier residues were omitted, and hydrogen atoms were added. After that, nonpolar hydrogens and lone pairs were merged and a Marsili-Gasteiger partial charge was assigned to each atom in the molecule. The coordinates of the grid box used for the blind docking technique were selected according to suggested active sites with maximum stability. Following the docking process, 10 conformations were generated for each target and ligand. The conformations were then evaluated based on their score (binding affinity), with the best conformation chosen according to its lowest negative energy and an RMSD of less than or equal to 2 angstroms.

### Molecular dynamics simulation studies

To validate the docking process, molecular dynamics simulations were conducted for a duration of 100 nanoseconds on monomers of Arabic gum. These simulations were carried out utilizing NAMD2 and VMD (version 1.9.3).

### Drug scanning

The druglikeness of monomers of Arabic gum was assessed following the Lipinski’s rule of five (http://www.scfbio-iitd.res.in/software/drugdesign/lipinski.jsp). The Lipinski Rule of Five is useful for identifying compounds that are drug-like and those that are not. It forecasts high probability of success or failure, due to the similarity of the molecules to drugs and their compliance with two or more of the following rules: molecular mass (< 500), hydrogen bond donors (≤ 5) and acceptors (≤ 10), log *P* value (≤ 5) and molecular refractive index (40–130) [26, 27].

### Statistical analysis

The data were analyzed using analysis of variance (ANOVA) (SPSS 18.0) followed by the Tukey post-hoc test. Sample values with *P* ≤ 0.05 and *P* ≤ 0.01 were considered statistically significant and highly significant, respectively.

## Results

### Characterization of the nanoparticles

**Fig. 1:**
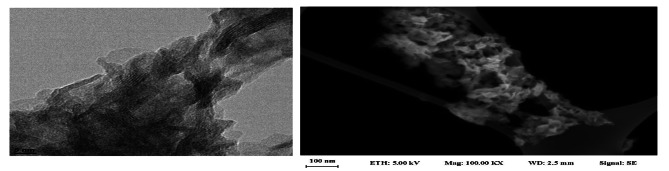
TEM images of MnO_2_-NPs (**a**) and FE-SEM image of MnO_2_-NPs (**b**)

The TEM and SEM images of the green-synthesized MnO_2_-NPs are shown in Fig. 1 (a,b). MnO_2_-NPs were synthesized by a green method using Arabic gum both as a template and reducing agent. MnO_2_-NPs were prepared by reducing potassium permanganate in the presence of Arabic gum. The MnO_2_-NPs were also extra thin wrinkle-like nanosheets. The average lateral dimension of MnO_2_-NPs is lower than 100 nm with the ultrathin thickness of nearly 5nm.

### Cytotoxicity of the green-synthesized MnO_2_-NPs

**Fig. 2:**
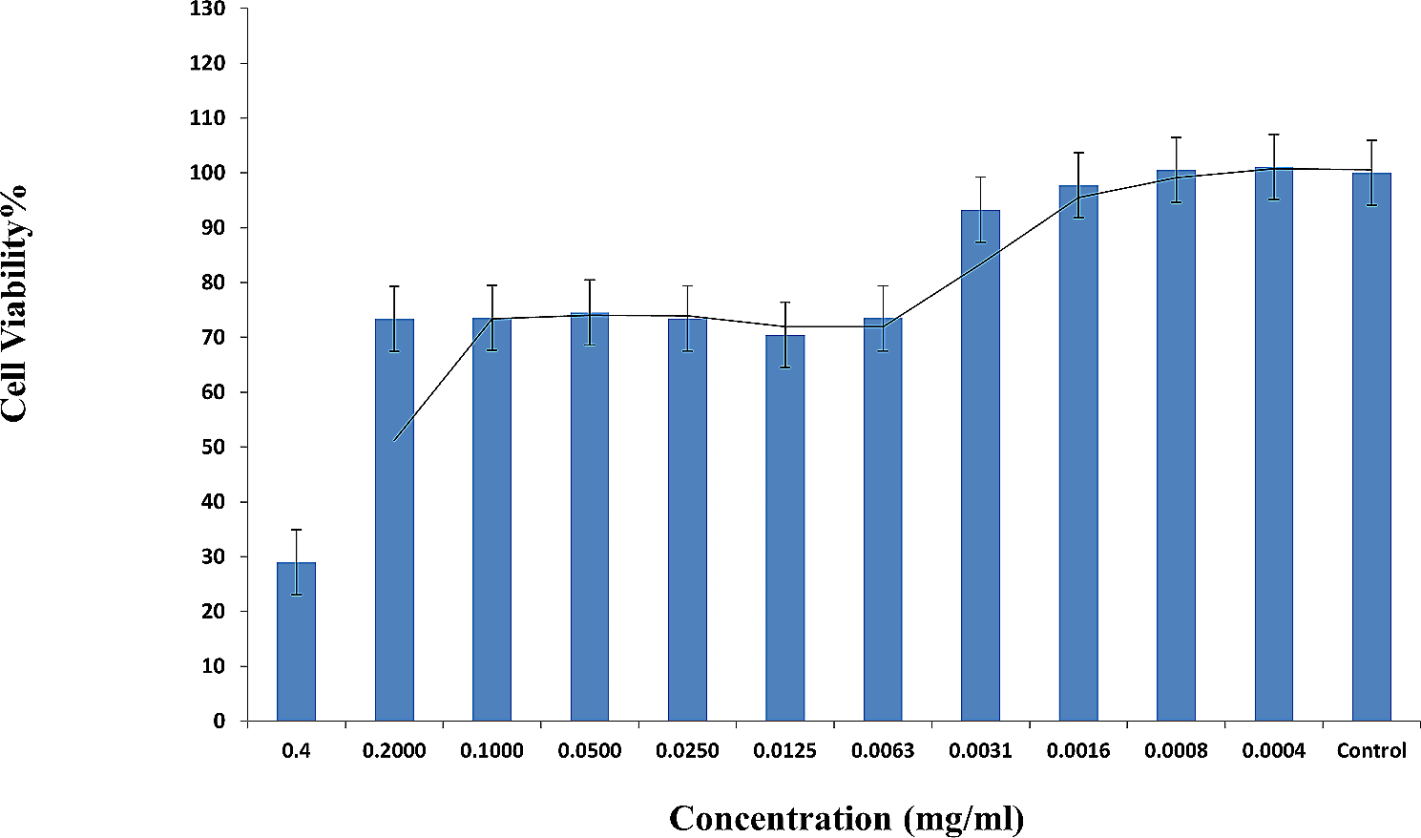
Cytotoxicity analysis of various concentrations of MnO_2_ on MDCK cells by an MTT assay

During the MTT cytotoxicity assay, it was determined that the green-synthesized MnO_2_-NPs had CC50 and NCTC values of 379 and 95 μg/mL, respectively (Fig. 2). Amantadine hydrochloride and oseltamivir carboxylate had CC50 values of 197 μg/ml and 788 μg/ml, respectively, when tested on MDCK cells. Additionally, NCTC values of 99 and 394 μg/ml were used for amantadine and oseltamivir, respectively.

### Antiviral activity of green synthesized Arabic gum-MnO_2_-NPs against influenza infection

**Fig. 3:**
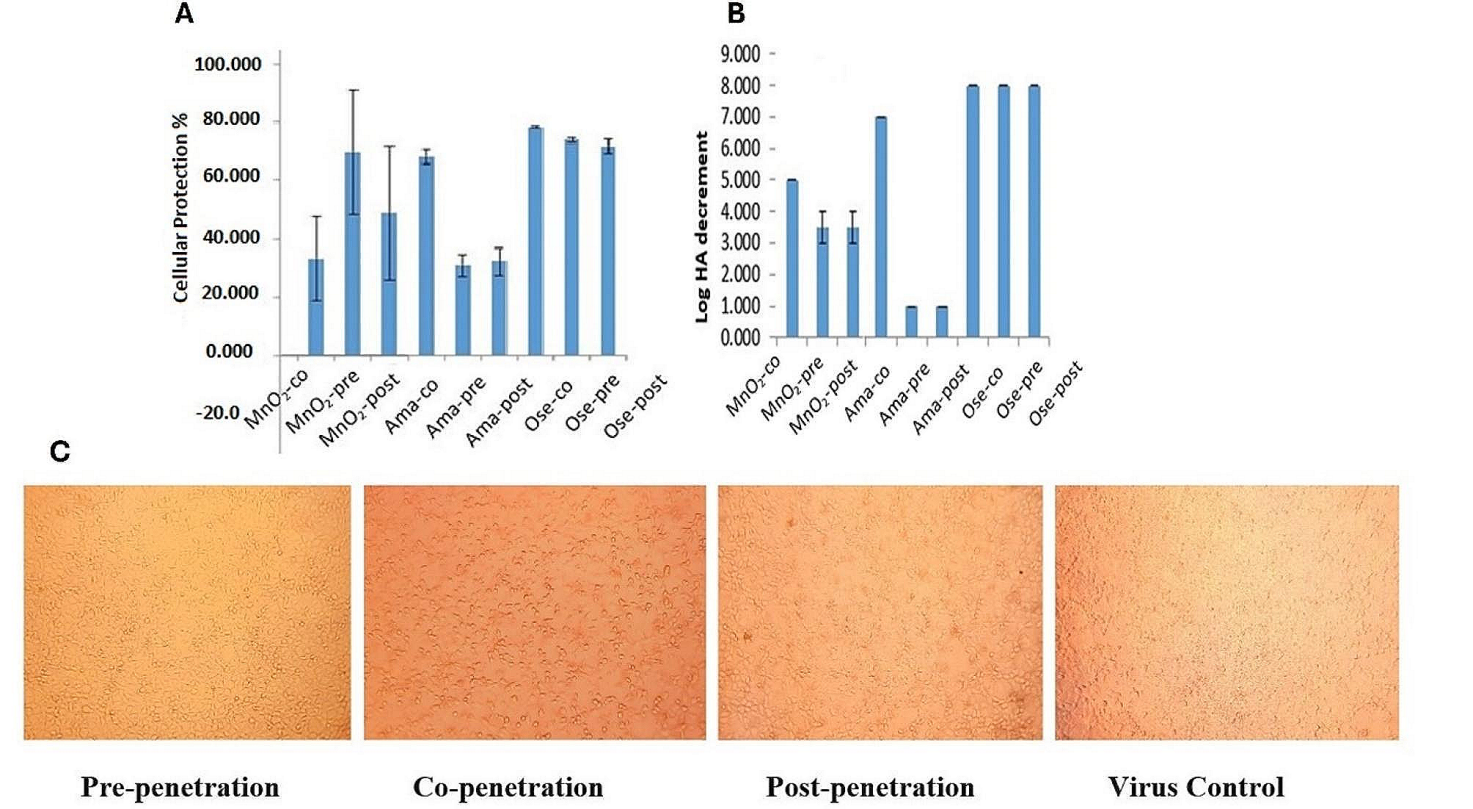
Dose-dependent antiviral response. Compound induced cellular protection % (**a**) and Log HA decrement (**b**). Cytopathic effects of *Arabic gum-MnO*_*2*_*-NPs* on influenza adsorption in MDCK cells. MDCK cells treated with the non-cytotoxic concentration (NCTC) dose of *Arabic gum-MnO*_*2*_*-NPs* in three modes (pre-, co-, and post-penetration of influenza virus). Cytopathic effects evaluated 48 h post-infection (c)

The antiviral capacity of the Arabic gum-MnO_2_-NPs was evaluated by determining both the viral titer and the cellular percentage of protection in pre-, co-, and post-treatments. The effect of the compound on viral titer was measured by decrements in the Log HA titer (Fig. 3B). Pre-penetration treatment with MnO_2_ resulted in a 3.5 log HA decrement (*p*-value < 0.01) and 69.7% cellular protection (*p*-value < 0.01), which demonstrated the most significant difference in cellular protection compared to other treatment and virus control group. Additionally, exposure of cells to NPs, both co- and post-exposure, resulted in significant decreases in HA titer (*p*-value < 0.01) compared to the virus control group. Furthermore, the percentage of protection in both co- and post-treatments was significantly higher, although lower than the pre-treatment level, when compared to the virus control group (*p*-value < 0.01). The results indicated that the compound had a preventive and protective effect on the cell viability against viral cytopathic effects, especially in pre-infection treatment. The cellular protection (A) and HA (B) results are illustrated in Fig. 3 with amantadine and oseltamivir being used as control antivirals in parallel. Notably, the cellular protection against viral control was 20%. Furthermore, as shown in Fig. 3C, H1N1-induced cytopathic effects were significantly decreased in the treated groups, especially in the pre-penetration group, compared to the control group.

### Molecular docking

**Fig. 4:**
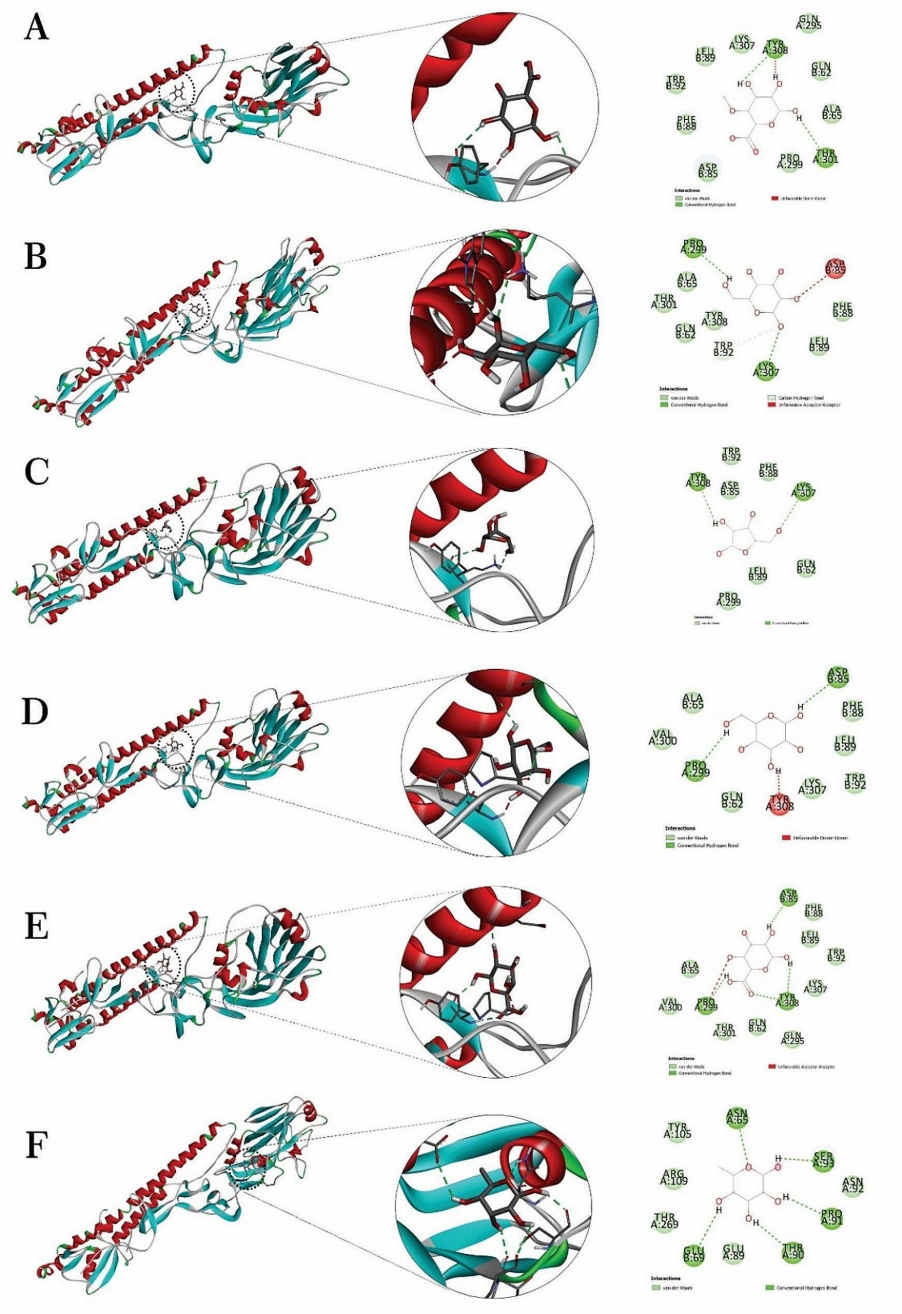
3D structures obtained through molecular docking related to complexes of target with **A**: 4-O-methyl-beta-D-glucuronic acid; **B**: alpha-D-galactopyranose; **C**: alpha-L-arabinofuranose; **D**: beta-D- galactopyranose; **E**: beta-D-Galactopyranuronic acid; **F**: L-rhamnopyranose

Autodock Vina was used for molecular docking, which resulted in 10 conformations for the interaction between each ligand and target. The best conformations were selected based on their minimum negative energy, ΔG (kcal/mol). The selected conformations and their corresponding binding affinities were listed in Table 1. The binding affinities were between − 3.3 and − 5.8 kcal/mole, relating with the interaction between the target with MnO_2_ and beta-D-galactopyranuronic acid, respectively. Figure 4 shows the intermolecular interactions of monomers of Arabic gum with target.

**Table 1:** Binding affinities of different selected conformations

Ligand	Binding Affinity
beta-D-Galactopyranuronic acid	-5.7
4-O-methyl-beta-D-glucuronic acid	-5.6
beta-D- galactopyranose	-5.5
alpha-D-galactopyranose	-5.3
L-rhamnopyranose	-5.1
alpha-L-arabinofuranose	-4.8
Manganese Dioxide	-3.3

### Molecular dynamic

**Fig. 5:**
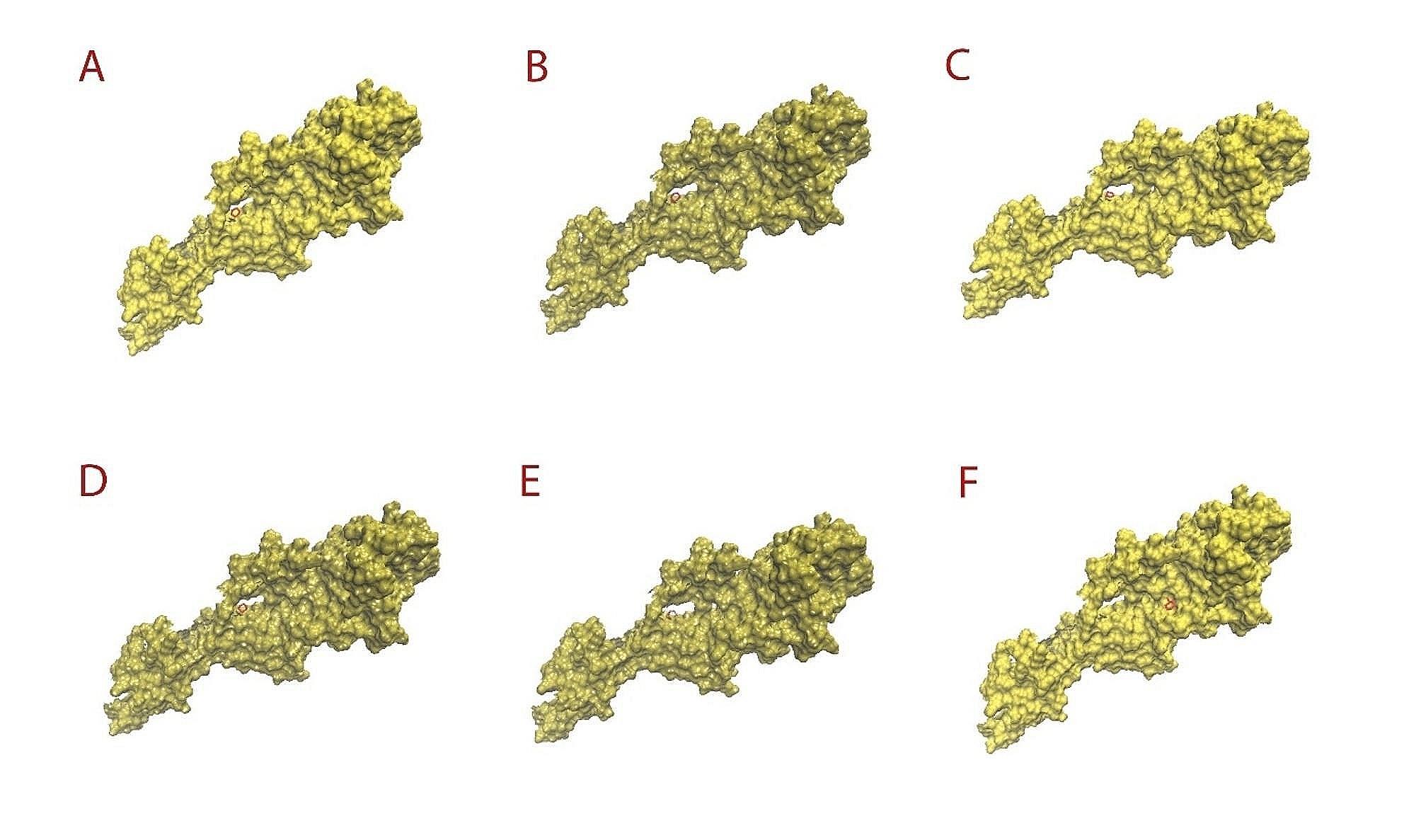
3D structures obtained through molecular dynamic related to complexes of target with **A**: 4-O-methyl-beta-D-glucuronic acid; **B**: alpha-D-galactopyranose; **C**: alpha-L-arabinofuranose; **D**: beta-D- galactopyranose; **E**: beta-D-Galactopyranuronic acid; **F**: L-rhamnopyranose

Monomers of Arabic gum were subjected to molecular dynamics. 3D structures of complexes with target obtained through molecular dynamics are shown in Fig. 5, which are in good agreement with the results obtained from molecular docking. Additionally, RMSDs listed in Table 2 demonstrate the stability of complexes during 10 runs of molecular dynamic simulation. A lower RMSD value indicates a greater stability of the protein complex. As observed, the RMSDs are in the range of 0.342–0.869 Å demonstrating all complex of monomers of Arabic gum with target are stable. This finding is also in accordance with those predicted through the docking simulations.

**Table 2:** RMSDs of complexes obtained from molecular dynamics

	Avg	SD	min	max
beta-D-Galactopyranuronic acid	0.514	0.130	0.342	0.658
4-O-methyl-beta-D-glucuronic acid	0.536	0.099	0.391	0.684
beta-D- galactopyranose	0.538	0.104	0.397	0.703
alpha-D-galactopyranose	0.601	0.127	0.412	0.790
L-rhamnopyranose	0.659	0.156	0.422	0.869
alpha-L-arabinofuranose	0.715	0.123	0.486	0.882

### Drug scanning

The drug screening findings presented in Table 3 indicated that Lipinski’s rule of five was satisfied by the Arabic gum monomers.

**Table 3:** Evaluation of monomers through five rules of Lipinski

Ligand	MW	HBD	HBA	LogP	A
beta-D-Galactopyranuronic acid	194	5	7	-0.071360	36.069992
4-O-methyl-beta-Dglucuronic acid	208	4	7	-2.475001	41.326191
beta-D- galactopyranose	180	4	5	0.272440	37.607491
alpha-D-galactopyranose	221	5	6	0.574620	49.221893
L-rhamnopyranose	164	4	5	0.572240	36.728691
alpha-L-arabinofuranose	150	4	5	0.255450	31.316692

## Discussion

Today, the focus of modern NP synthesis is increasingly shifting towards green synthesis methods, attributed to their numerous advantages over conventional methods. Notably, green synthesis methods are eco-friendly as they avoid the use of toxic chemicals, making them highly desirable for the production of NP-based antiviral agents [28]. MnO_2_-NPs, known for their remarkable stability and immunogenicity, are emerging as potent candidates in the realm of antiviral therapies [29]. Arabic gum, a complex polysaccharide consisting of an extremely branched arrangement of D–glucuronic acid, L-arabinose, D-galactose, and L-rhamnose linked to flavonoids [30, 31], provides a sustainable and efficient medium for the green synthesis of MnO_2_-NPs. This method is gaining recognition as a promising approach for developing new antiviral agents.

In this research, we embarked on the pioneering investigation of the antiviral activity of Arabic gum-MnO_2_-NPs, specifically targetingH1N1 replication in an in vitro system. The results of our study demonstrate that a nontoxic concentration of Arabic gum-MnO_2_-NPs can reduce the production of H1N1 progeny in the MDCK cell line. The Arabic gum-MnO_2_-NPs treatment led to a dose-dependent reduction in H1N1 infectivity. The cytotoxicity of these NPs was determined by treating the MDCK cell line using varying concentrations of the NPs. Based on the cytotoxicity assay, the CC50 and NCTC of the Arabic gum-MnO_2_-NPs were 379 and 95 μg/mL, respectively.

Over recent years, several studies have been conducted to assess the inhibitory properties of different green synthetized nanostructures on drug-resistant viral infections [32–34]. For instance, Rabiee et al. synthesized green ZnO-NPs for the first time using the extract from Salvia hispanica leaves, in which anti-influenza activity was found to be promising [35]. Natural products such as Arabic gum as reducing and stabilizing agents have exclusive properties to enhance the biocompatibility and biodegradability of the resulting NPs [36]. The antiviral activity of Arabic gum NPs is attributed to their ability to inhibit the replication of viruses by interfering with their entry, attachment, and fusion with host cells [36]. There are limited studies performed on the effect of Arabic gum on viral infection. In a study, Ghobashy et al. assessed the antiviral capacity of Arabic gum by reduction of the binding affinity of SARS-CoV-2 (RBD) and the angiotensin-converting enzyme 2 (ACE2). They used fluorometric assay to investigate the probability of Arabic gum and ACE2 interactions. The outcomes confirmed that the interaction could take place between Arabic gum and ACE2 and can reduce SARS-CoV-2 entry [23]. In another study by Eladi et al., following vitamin C and Arabic gum co-administration, significant changes were found in hemagglutinin inhibition antibody titers, clinical signs, mortality rates, gross lesions, virus shedding rates, para-clinical parameters and lung lesions scores. Therefore, they suggested that such a combination can provide protective outcomes against influenza H9N2 infection [37].

In accordance with these results, our study showed that the Arabic gum-MnO_2_-NPs have inhibitory activities to reduce the penetration of influenza virus effectively, when added before infection. Also, it was demonstrated that the Mn^2+^ released by MnO_2_-NPs plays an important part in regulating the immune response during viral diseases [38]. When a virus infects a host cell, the cell releases Mn^2+^ from its membrane organelles into the cytosol. This Mn^2+^ accumulates and triggers the cGAS-STING signaling pathway, leading to the phosphorylation of IRF3, activation of the NF-κB pathway, and stimulation of IFN-I production and ultimately results in antiviral effects [38]. Our results demonstrated that the Arabic gum-MnO_2_-NPs have antiviral activity in MDCK cells, possibly through blocked particular receptors involved in attachment and entry of influenza particles into the host cells. Moreover, experimental data revealed that the Arabic gum-MnO_2_-NPs target and interfere with different stages in the proliferation cycle of the influenza virus and this outcome could be Arabic gum-MnO_2_-NPs direct effect or the effects of activated immune response.

As the results indicated, both post- and co-exposure of cells to MnO_2_-NPs led to a significant reduction in the titer of the influenza virus, although these effects were less pronounced than those observed in pre-exposure. Additionally, in the treatments involving Arabic gum-coated MnO_2_-NPs, both pre-penetration and post-penetration, the inhibitory effect was significantly greater than the amantadine control, yet lower in comparison to the oseltamivir control group. These variations may stem from the distinct functions of green-synthesized MnO_2_-NPs in infected cells compared to the drug control groups. Therefore, a more comprehensive understanding of the precise antiviral mechanisms of MnO_2_-NPs at each stage of the virus replication cycle, through detailed studies, can enhance the clarity of this issue. Similarly, Li et al. investigated the antiviral capabilities of selenium nanoparticles (SeNPs) and oseltamivir surface-modified SeNPs (Se@OTV) against H1N1 influenza virus. Their findings indicated that Se@OTV had higher antiviral activity and was less toxic. It was suggested that the mechanism of action may involve interference with the influenza virus life cycle by inhibiting hemagglutinin and neuraminidase activities. During the final stage of influenza virus replication, the neuraminidase enzyme cleaves the attachment between hemagglutinin on the virus and the sialic acid receptor on the host cell. Oseltamivir is a sialic acid analogue and a neuraminidase inhibitor that prevent this cleavage step, interfering with the release of progeny influenza virus from infected host cells and preventing infection progression. The use of oseltamivir-modified SeNPs in this study suggests that inhibition of hemagglutinin and neuraminidase activities may be the possible antiviral mechanism [39].

Besides the antiviral test, the results of docking were promising. In this regard, an interaction with a lower ΔG (kcal/mol) is more stable, so a ligand-receptor conformation with the lowest binding affinity is more acceptable. The binding affinity values under the − 4 were assigned to stable interaction between ligand and receptor. In the present study, nearly all monomers of Arabic gum showed a good affinity to targets. Although, beta-D-galactopyranuronic acid and 4-O-methyl-beta-D-glucuronic acid revealed better affinity to the target that can be attributed to their -HCOO group. Moreover, as expected MnO_2_ showed no interaction with target.

There were some limitations in this study. Firstly, it may be beneficial to evaluate the anti-influenza effect of Arabic gum and MnO_2_ separately, necessitating further study. Secondly, the particular mechanism underlying the antiviral activity and immunogenicity of the green synthetized MnO_2_ in the present study remain unknown, highlighting the need for additional research in the future.

## Conclusions

This study is the first report of Arabic gum-MnO_2_-NPs and their inhibitory activity in influenza infection. The present study suggests that Arabic gum-MnO_2_-NPs, synthesized through an environmentally friendly process, may serve as effective anti-influenza agents. They appear to obstruct the virus by blocking its receptors and hindering cell penetration. Although treatments applied during and after virus penetration significantly reduced virus titer, further research is required to elucidate the precise underlying mechanisms. The green synthesis of MnO_2_-NPs using Arabic gum is a promising approach for the production of antiviral agents. This method stands out for its simplicity, cost-effectiveness, and minimal environmental impact. The resultant MnO_2_-NPs demonstrate antiviral activity. However, further research is needed to explore the full potential of these NPs as antiviral agents, potentially opening new avenues in the fight against influenza and other viral diseases.
